# Comparison of axial and coronal acquisitions by non-contrast-enhanced renal 3D MR angiography using flow-in time-spatial labeling inversion pulse

**DOI:** 10.1007/s10334-019-00796-6

**Published:** 2019-12-28

**Authors:** Junji Takahashi, Yuki Ohmoto-Sekine, Takashi Yoshida, Mitsue Miyazaki

**Affiliations:** 1 Radiology Department, Toranomon Hospital, 2-2-2 Toranomon, Minato-ku, Tokyo, 105-8470 Japan; 2 Health Management Center, Toranomon Hospital, 2 2-2 Toranomon, Minato-ku, Tokyo, 105-8470 Japan; 3 Department of Radiology, University of California, San Diego, 9452 Medical Center Drive, La Jolla, CA 92037 USA; 4 Canon Medical Systems, Corp., Tochigi, Japan

**Keywords:** Non-contrast-enhanced MRA: renal artery, Time-spatial labeling inversion pulse (Time-SLIP), Renal MRA screening, Flow-in technique

## Abstract

**Objective:**

We evaluated image quality differences between axial and coronal non-contrast-enhanced renal three-dimensional (3D) magnetic resonance angiography (MRA) acquisitions, using time-spatial labeling inversion pulse (Time-SLIP) with flow-in balanced steady-state free precession (bSSFP).

**Materials and methods:**

Axial and coronal images were acquired in 128 subjects using non-contrast-enhanced 3D-MRA with Time-SLIP flow-in bSSFP on a clinical 1.5-T MRI system. Visualization of source and maximum intensity projection (MIP) images of renal arteries were compared between the axial and coronal acquisitions using a four-point scale. For quantitative analysis, vessel-to-background contrast ratios of aorta and renal arteries were calculated.

**Results:**

Both acquisitions yielded similarly excellent quality. In source image evaluation, coronal acquisitions showed significantly more motion degradation (*p* < 0.01) than did axial acquisitions. In MIP image evaluation, coronal acquisitions yielded superior image quality, less motion degradation, and better visualization of the number of renal branches than did axial acquisition. The renal artery to background signal contrast was greater in coronal than in axial acquisitions (*p* < 0.01).

**Conclusion:**

Coronal acquisition provides superior contrast between the renal arteries and background and allows more persistent visualization than axial acquisitions in non-contrast-enhanced MRA using flow-in bSSFP with Time-SLIP. First-line screening of renal non-contrast-enhanced MRA should involve coronal acquisition.

## Introduction

Based on the consensus of the American College of Cardiology Foundation/American Heart Association (ACCF/AHA) guidelines and European Society of Cardiology (ESC) guidelines, renal magnetic resonance angiography (MRA) has been established as a class IB diagnostic tool for the screening of renal artery stenosis (RAS), despite its tendency for overestimating the degree of luminal narrowing [1, 2]. Non-contrast-enhanced MRA techniques have been gaining interest because gadolinium-based contrast agents (GBCA) may cause nephrogenic systemic fibrosis (NSF) in patients with renal insufficiency [3].

In addition, there is a concern about the risks related to toxicities and deposition of GBCA in various tissues [4], particularly the brain [5, 6]. Thus, non-contrast-enhanced MRA techniques, using spin-labeling with three-dimensional (3D) balanced steady-state free precession (bSSFP), have been developed and applied in many contexts [7–10]. A technique for renal artery assessment using 3D bSSFP has been reported as a safe and effective approach for the evaluation of RAS [11–15]. Utsunomiya et al. [16] have reported that non-contrast-enhanced MRA using time-spatial labeling inversion pulse (Time-SLIP) with 3D flow-in bSSFP provides a non-invasive and effective method for evaluating the degree of stenosis in renal arteries against contrast-enhanced computer tomography angiography (CTA) or digital subtraction angiography (DSA). Albert et al. [17] have compared Time-SLIP with CTA and found that non-contrast approach has provided equivalence in determining the presence or absence of RAS in an international multi-center study.

Although the axial acquisition with higher in-plane resolution is proficient for detecting RAS, the coverage of the axial acquisition is limited due to the number of slices in 3D slab within a reasonable scan time. An axial coverage in the body’s superior–inferior or Z-direction requires longer scan times and limits visualization of various other vessels located outside the Z-direction coverage. In contrast, the coronal acquisition has the benefit of wide coverage of the body in the superior–inferior direction, with the slice or 3D slab direction in the anterior–posterior direction of the body. Additionally, we had evaluated coronal acquisitions via Time-SLIP using a short tau inversion time (STIR) pulse, and found that it provided superb renal MRA images in healthy volunteers [18]. The application of the STIR pulse allows large coronal coverage with a uniform background and fat signal suppression, as compared to chemical-selective or frequency-selective fat suppression (CHESS) when using 3D bSSFP read-out.

Various applications of Time-SLIP with flow-in 3D bSSFP have been used for visualization of abdominal vessels, including the renal and hepatic arteries, and the portal veins [19]. In particular, in renal MRA, application of the Time-SLIP flow-in technique was further optimized in terms of the inversion time (TI) nulling point [20]. When the TI is near the null point of the kidney parenchymal signal, the best contrast between renal artery and background signals is obtained. In an evaluation of renal Time-SLIP flow-in in non-contrast-enhanced MRA, Parienty et al. studied severe RAS using images acquired in both coronal and axial directions, and compared these with DSA images [21]. In their evaluation, both acquired directions were used to measure the degree of stenosis. However, two axial and coronal directional scans take over 10 min and, therefore, it is not realistic to perform both scans in daily clinical practice. If only one renal scan is permitted within the examination time, what is the first choice of non-contrast-enhanced renal MRA scan between the axial and coronal acquisitions? Our purpose is to compare the advantages and disadvantages between the axial and coronal direction acquisitions of non-contrast-enhanced renal MRA using the flow-in Time-SLIP 3D bSSFP.

## Materials and methods

### Subjects

This retrospective study was approved by the institutional review board (IRB) of our hospital and informed consent was obtained from all subjects. This retrospective study was performed on 128 subjects (88 men and 40 women, age range 29–85 years, average 62.1 ± 11.1 years) for evaluation by non-contrast-enhanced real artery MRA screening at Toranomon Health Management and Diagnostic Imaging Center (Tokyo, Japan) enrolled from January through September of 2015.

### MRI protocol

All MRA examinations were performed with a commercial 1.5-T scanner (Canon Medical Systems Corp., Tochigi, Japan) equipped with a 16-element ATLAS body-coil. Non-contrast-enhanced MRA was performed using segmented 3D imaging with a flow-in bSSFP sequence, with Time-SLIP. The Time-SLIP pulse utilized a slice-selective IR (sIR) pulse that inverts all spins of the tagged region to − 180° in the longitudinal magnetization (− Mz). During the TI interval, the spins exponentially recover via T1-relaxation, and untagged fresh blood travels from the aorta into the tagged region, where the renal arteries are located. The bSSFP sequence consists of a preparation pulse, dummy pulses, and fully balanced gradients in all three directions, which provide a high signal-to-noise ratio (SNR) in bright-blood images, due to intrinsic T2/T1 contrast. The detailed pulse sequence diagram and the relationship between the untagged blood and tagged region with magnetization state are shown in Ref. [19] and Fig. 4a.

Prior to these studies, we had optimized the axial acquisition using this technique by means of varying TI times, flip angle, and the number of segmentations for fat suppression were performed to depict the renal artery branches, within a reasonable scan time, which has been used in these studies [22].

**Fig. 1:**
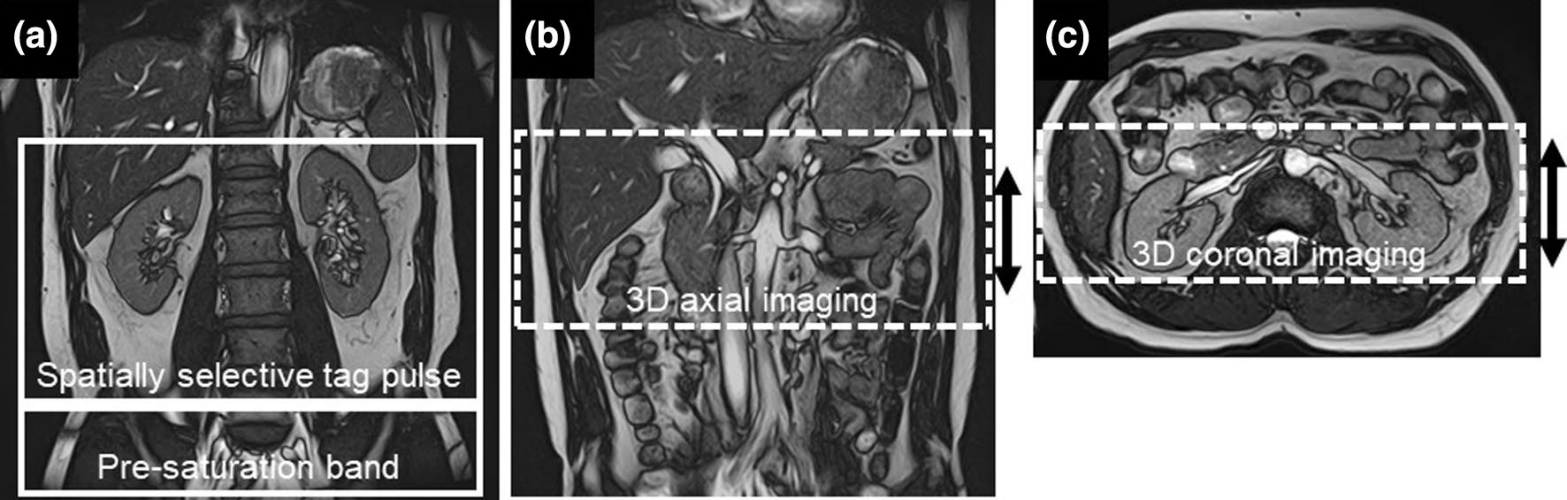
The representative imaging plan for Time-SLIP pulse (tag pulse), axial and coronal acquisitions. **a** Coronal 2D scout image shows the position of kidneys. The white solid frame represents the position of the Time-SLIP pulse, which was placed immediately above the superior poles of both kidneys, and white box is a pre-saturation band pulse placed below the Time-SLIP pulse. **b** Coronal 2D scout image shows abdominal aorta to renal arteries. The white dotted frame represents the position of axial acquisition slab (arrow) in the superior–inferior direction. **c** Axial 2D scout image shows renal arteries and veins. The white dotted frame represents the position of coronal acquisition slab (arrow) in the anterior–posterior direction. The Time-SLIP pulse and pre-saturation band pulse are placed similarly in both axial and coronal acquisitions

Before each examination, we have instructed all subjects to have regular breathing and remain awake throughout the experiment. We also used an abdominal respiration belt in all subjects, and wrapped around the abdomen to control their expiration and suppress breathing-related motion of the abdomen [23]. The abdominal aorta and renal arteries were first localized using two-dimensional (2D) bSSFP coronal breath-hold scout imaging and then the axial breath-hold scout imaging with the following parameters: repetition time/echo time (TR/TE) = 4.4/2.2 milliseconds (ms), flip angle = 70°, matrix = 256 × 256, field-of-view (FOV) = 35 cm × 35 cm, slice thickness (ST) = 6 mm, number of slices = 16, and acquisition time (AT) = 16 s, without applying fat suppression. Following the 2D scout imaging, a spatial slice-selective Time-SLIP pulse (slice thickness of 240 mm) with a TI of 1500 ms was applied in the transverse plane. In most cases, the Time-SLIP pulse was placed immediately above the superior poles of both kidneys and was large enough to cover the vasculature of interest (aorta to renal arteries). A pre-saturation band pulse was applied to reduce the undesired signal of inflowing blood from the inferior vena cava. The position of the Time-SLIP pulse was graphically localized to suppress signals from background tissues within the imaging volume, as shown in Fig. 1. Data acquisition was accelerated with a parallel imaging (PI) factor of two in the phase-encoding (PE) direction. Depending on the acquired number of imaging sections and TI, the axial acquisition time ranged from 4.2 to 13.0 min and the coronal acquisition time ranged from 3.2 to 13.8 min. The imaging parameters were as follows: TR/TE = 4.3/2.2 ms, flip angle = 120°, matrix = 256 × 256 (interpolated to 512 × 512), TI = 1500 ms, FOV = 35 cm × 35 cm, 3-mm axial section slices (interpolated to eighty 1.5-mm section slices, resolution of 0.68 × 0.68/1.5 mm) or 2.5-mm coronal slices (interpolated to eighty 1.25-mm section slices, resolution of 0.68 × 0.68/1.25 mm), PI = 2.0, Time-SLIP tag-slice thickness = 240 mm, and segmentation of k-space = 2, using respiratory gating. The axial acquisition was performed using a CHESS fat-suppression technique and the coronal acquisition was performed using the STIR pulse with an inversion duration of 190 ms. The coronal 3D bSSFP acquisition required uniform fat suppression in a large coverage in the Z-direction without having banding artifacts, whereas the axial 3D bSSFP acquisition required a relatively smaller *Z*-directional coverage, and CHESS worked well.

### Data analysis

All MR images were transferred to a workstation (Zio station2; Ziosoft Corporation, Tokyo, Japan) for post-processing. Maximum image projection (MIP) images for the entire volume of the renal artery were obtained by an experienced radiological technologist with standardized post-processing procedures for both axial and coronal acquisitions. Entire volumetric MIP images were generated by rotating the 3D data set through 180° at 15° increments for assessing renal arteries. Source images were also used for this evaluation.

Image review and analysis were performed independently by two readers (a radiological technologist with 25 years of experience in MRI and a cardiologist with 10 years of experience in cardiovascular imaging). Both readers were blinded to the patient’s clinical information. Image quality was evaluated for both source and MIP images for renal arteries. Each artery was rated on a four-point scale: grade 3, excellent with high homogeneous signal intensity within the vessel lumen and good delineation of the vessel border, and no artifacts present; grade 2, good visualization of the vessel lumen, incomplete delineation of the vessel border, although some artifacts may be present; grade 1, fair visualization with low, inhomogeneous signal intensity, incomplete delineation of the vessel border, and diagnosis may be impaired; and grade 0, poor visualization and diagnosis not possible. Image quality scores of 2 or above were defined as acceptable image quality. The images were also assessed on a four-point scale for motion degradation at the renal arteries and branches: grade 3, no visible motion degradation; grade 2, minimal motion degradation; grade 1, moderate motion degradation with blurring of the vessel border, but diagnostic; and grade 0, severe motion degradation and non-diagnostic. In quantitative assessment, the number of branch vessels was assessed by counting on MIP images.

Region of interest (ROI) analysis was performed on the renal arterial signal intensity (SIA) and renal cortex, renal medulla, and muscle as background signal intensities (SIB) for both coronal and axial acquisitions. A ROI with 14.02 mm^2^ area was placed manually on the renal artery and background. The relative signal contrast ratio between the artery and background signals, i.e., vessel-to-background ratio (VBR), VBR = (SIA − SIB)/SIA were measured from the source images.

### Statistical analysis

Statistical analyses were performed using commercially available software, Statistical Package for Social Science (SPSS) for Windows, ver.13.0 (Chicago, IL USA).

Data were presented as means ± standard deviations. Paired Student’s *t* test was used to evaluate the contrast ratio. Wilcoxon’s signed-rank test was used for comparison of image quality, motion degradation, and the number of countable renal arterial branches. A *p* value of less than < 0.05 was considered to indicate a statistically significant difference.

The inter-reader agreement was calculated using Cohen’s kappa statistics with the following interpretation: poor, < 0.4; good, ≥ 0.4 and < 0.75; excellent, ≥ 0.75. We use same metrics (Cohen’s kappa) for the assessment of inter-reader agreement with both image quality and motion degradation. Reviewer scores were averaged, and statistical analyses of image quality and motion degradation were performed on both source and MIP images for each renal artery. We adopted the image score of reader 1 and compared inter-reader agreement with reader 2.

## Results

**Fig. 2:**
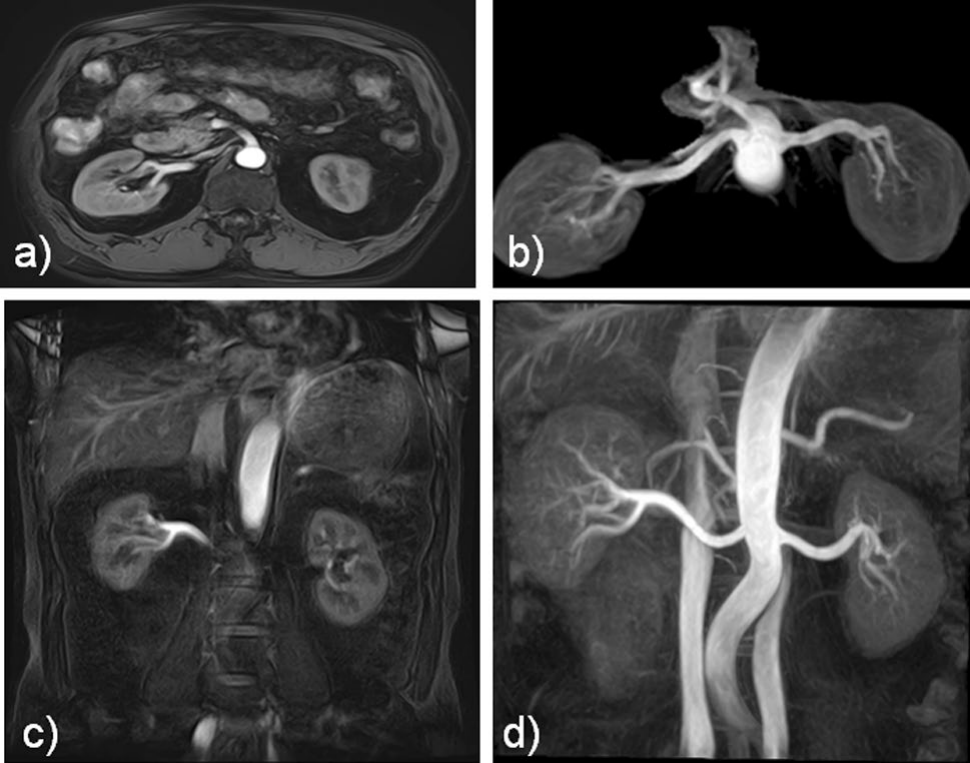
The source and maximum intensity projection (MIP) images of a 62-year-old male who underwent vascular screening. **a** Axial source image, **b** axial MIP image, **c** coronal source image, and **d** coronal MIP image. Motion degradation can be seen in the distal segment of the right renal artery branches

**Fig. 3:**
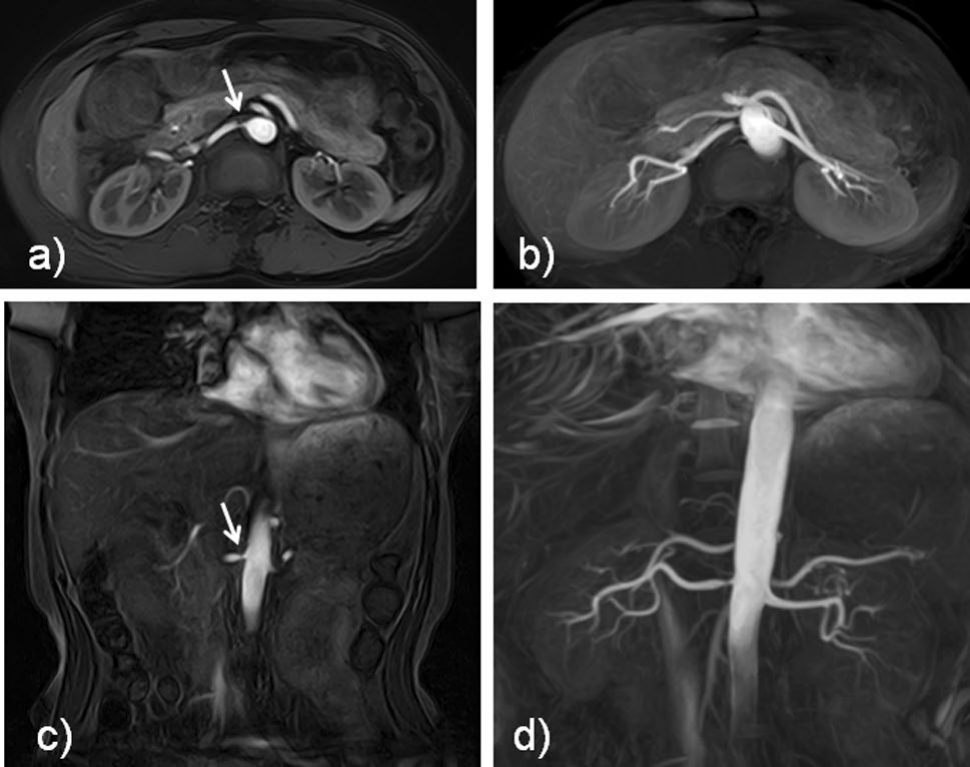
The source and maximum intensity projection (MIP) images of a 58-year-old female with right renal artery stenosis. **a** Axial source image, **b** axial MIP image, **c** coronal source image, and **d** coronal MIP image. Both **a** axial and **c** coronal source images show the right renal stenosis (arrows)

**Fig. 4:**
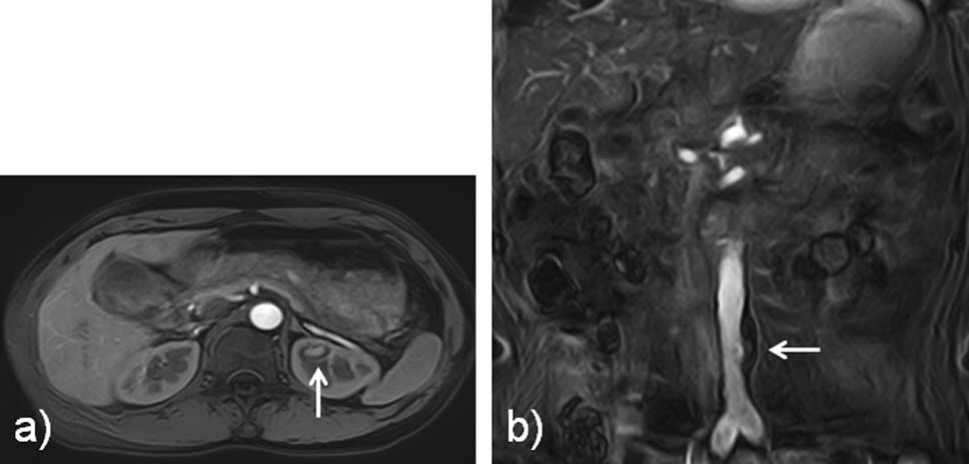
Additional information on these cases. **a** The source image of a 49-year-old male with an intrarenal hemorrhage in the left kidney (arrow), seen on the axial acquisition. **b** The source image of a 76-year-old female with an abdominal aortic aneurysm, seen on the coronal acquisition. Note that an intramural thrombus is present in the aneurysm (arrow)

Both axial and coronal flow-in bSSFP images with well-depicted renal arteries were successfully obtained in all 128 subjects. Two subjects underwent nephrectomy (one on the right, the other on the left). Two hundred and fifty-four renal arteries were evaluated in this analysis. Representative images are shown in Figs. 2 and 3. In general, both axial and coronal acquisitions presented well-depicted renal arteries, using our methods (Fig. 2). In addition, both axial and coronal source images permitted the depiction of right renal artery stenosis, as shown in Fig. 3. In addition, Fig. 4 shows interesting cases. Imaging scan time was 6.6 ± 1.4 min (mean ± SD; range 4.2–13.0 min) in the axial acquisition, and 6.5 ± 1.8 min (mean ± SD; range 3.2–13.8 min) in the coronal acquisition. There was no significant difference (*p* = 0.087) in the scan time between the acquisitions. The total scan time for both scans was approximately 30 min, including preparation scans, such as 2D scout imaging, using a single 16-s breath-hold acquisition for the axial and coronal scans.

### Qualitative analysis

Visual evaluation of source and MIP images of renal arteries between the axial and coronal acquisitions is presented in Table 1. For both the right and left renal arteries, there was no statistically significant difference in the image quality between the axial and the coronal acquisitions. The results of Cohen’s kappa statistics are summarized in Table 2. Inter-reader agreement of source image evaluation was excellent in right and left renal arteries on both axial acquisition and coronal acquisition. For MIP images, kappa values were also excellent in right and left renal arteries on both axial acquisition and coronal acquisition, indicating excellent inter-reader agreement.

**Table 1:** Comparison of image quality, motion degradation and visible number of branches between the axial and coronal acquisitions

Visual evaluation	Axial	Coronal	*p* value
Source image			
Rt renal A			
Image quality	2.87 ± 0.36	2.83 ± 0.39	0.35
Motion degradation	2.57 ± 0.56	2.32 ± 0.58	< 0.01*
Lt renal A			
Image quality	2.88 ± 0.35	2.83 ± 0.38	0.11
Motion degradation	2.66 ± 0.55	2.38 ± 0.59	< 0.01*
MIP image			
Rt renal A			
Image quality	2.70 ± 0.54	2.78 ± 0.47	0.12
Motion degradation	2.17 ± 0.66	2.33 ± 0.59	0.02*
Number of branches	3.88 ± 1.00	4.19 ± 1.01	< 0.01*
Lt renal A			
Image quality	2.72 ± 0.53	2.83 ± 0.39	0.02*
Motion degradation	2.40 ± 0.67	2.64 ± 0.56	< 0.01*
Number of branches	3.67 ± 0.93	4.22 ± 0.91	< 0.01*

*Rt* right, *Lt* left, *renal A* renal artery, *MIP* maximum intensity projection, *p < 0.05

**Table 2:** Inter-reader agreement of image quality and motion degradation of the axial and coronal acquisitions

Kappa statistic	Axial		Coronal	
	*κ*	95% CI	*κ*	95% CI
Source image				
Rt renal A				
Image quality	0.88	0.53–1.23	0.90	0.60–1.20
Motion degradation	0.84	0.58–1.10	0.86	0.62–1.10
Lt renal A				
Image quality	0.93	0.66–1.21	0.92	0.63–1.20
Motion degradation	0.95	0.80–1.10	0.94	0.79–1.11
MIP image				
Rt renal A				
Image quality	0.99	0.91–1.07	0.98	0.88–1.09
Motion degradation	0.91	0.75–1.08	0.90	0.70–1.09
Lt renal A				
Image quality	0.93	0.74–1.12	0.97	0.83–1.12
Motion degradation	0.93	0.79–1.07	0.92	0.74–1.11

*Rt* right, *Lt* left, *renal A* renal artery, *MIP* maximum intensity projection, *κ* kappa value, *Cl* confidence interval

In source image evaluation, the motion degradation of coronal acquisitions was significantly greater (*p* < 0.01) than that of the axial acquisitions. In MIP images, the motion degradation and the number of visualized branches were significantly greater than those of the axial acquisition (*p* < 0.01). We use the same metrics (Cohen’s kappa) for the assessment of the inter-reader agreement with motion degradation. The kappa statistics was interpreted as follows: poor < 0.4, good ≥ 0.4, and excellent ≥ 0.75.

### Quantitative analysis

The contrast ratio between the artery and background signals is shown in Table 3. The contrast ratios measured in the coronal acquisitions were significantly higher (*p* < 0.01) than those of the axial acquisitions.

**Table 3:** Comparison of contrast ratio between the axial and coronal acquisitions

Contrast ratio	Axial	Coronal	*p* value
Aorta vs muscle	0.77 ± 0. 03	0.89 ± 0.04	< 0.01*
Rt renal A vs muscle	0.74 ± 0.04	0.89 ± 0.04	< 0.01*
Rt renal A vs cortex	0.50 ± 0.09	0.69 ± 0.09	< 0.01*
Rt renal A vs medulla	0.68 ± 0.07	0.86 ± 0.09	< 0.01*
Lt renal A vs muscle	0.71 ± 0.06	0.88 ± 0.04	< 0.01*
Lt renal A vs cortex	0.46 ± 0.11	0.72 ± 0.09	< 0.01*
Lt renal A vs medulla	0.66 ± 0.09	0.86 ± 0.09	< 0.01*

*Rt* right, *Lt* left, *renal A* renal artery, *p < 0.01

## Discussion

The results of both axial and coronal acquisitions show successful well-depicted non-contrast-enhanced renal MRA images in all 128 subjects. Possible reasons of such a success can be as follows: (1) we utilized the abdominal respiration belt which maintains a regular respiration and decreases irregular respiration and deep breathing. This simple method was accepted by all subjects. (2) We acquired 2D scout images with both axial and coronal directions, which allow simple visualization and positions of aorta and renal artery bifurcation in each subject. We believe that 2D scout images with 2D bSSFP without fat suppression helped providing advance knowledge of renal and surrounding tissue positions, which makes easy and simple for setting 3D scan with the Time-SLIP pulse and pre-saturation pulse. If subjects breathe fast and constantly, the acquisition time will be short. On the other hand, when subjects sleep during a scan, the breathing cycle tends to be longer, and thus the acquisition time is longer.

We qualitatively and quantitatively analyzed flow-in Time-SLIP renal MRA images acquired in both axial and coronal acquisitions. The evaluation was performed not only on the MIP images, but also on the source images. We found that coronal acquisitions provided superior results compared to the axial acquisition in all analyses except for motion degradation in source images. In the qualitative analysis, the number of visualized branches in the MIP images of coronal acquisitions was greater than that of axial acquisitions, due to a higher contrast ratio and spatial resolution in the coronal acquisition. However, the results of motion degradation findings were contradicted between the source images and the MIP images. This contradiction could be explained as follows: (1) There is an error in the respiratory synchronous trigger, which often occurs when patients undergo changes in respiratory rates during acquisition, e.g., when patients fall asleep during acquisition, and in patients with respiratory disease. (2) Breathing-related movement of the kidneys occurs in a superior–inferior or head–foot direction; therefore, the coronal acquisition demonstrates kidney displacement due to respiration and motion degradation is slightly increased. However, in the MIP process, higher signals predominate in the MIP image; therefore, motion degradation of the coronal acquisition is minimized, and results are superior to those of source images, particularly in the distal branches of renal arteries.

In terms of quantitative analysis, our results indicated that the contrast ratio between arteries and background of coronal acquisitions was superior to that of axial acquisitions, which may be due to differences in fat saturation techniques. STIR suppresses not only fat signal but also the background signals. Shonai et al. [20] has reported that the STIR fat-suppression method provides better background signal suppression in the intestines and the parenchymal organs than does the CHESS method. Consequently, STIR provides higher contrast between the arteries and the background. As an application, Kanki et al. [24] has utilized an appropriate TI to differentiate cortex and medullar segments in the kidney.

The use of this technique at 3 T improves the blood-to-background signal, with higher contrast, simply due to a gain in signal and an intrinsically longer T1 value at 3 T than at 1.5 T, which helps to maintain suppressing background signals with longer TI or blood travel-time values of 1800–2000 ms [17].

Despite some minor disadvantages, the coronal acquisition using flow-in Time-SLIP renal MRA allows a wider coverage in the *Z*-direction or superior–inferior direction of the abdomen, and enable to observe the relationship between the renal arteries and accessory arteries, despite the position of the right and left kidneys. Other merits of the coronal acquisition are, for instance, that it facilitates a study of the relationship between the vessel lumen and wall in the irregularity of the aorta in the *Z*-direction, such as aneurysmal diseased lesions, visualization of the accessory renal arteries, and confirmation of the presence of any RAS in transplanted kidney patients with horizontally misaligned right and left kidneys. In the assessment of renal artery stenosis, the approach tends to overestimate the degree of luminal narrowing. In addition, the source image of axial acquisitions provides unambiguous visualization of the aorta, renal artery, and renal medulla/cortex on the same image. However, in cases where the right and left kidneys differ in the superior–inferior position, axial acquisitions will need to be extended due to the increase in the number of slices required.

Our study had several limitations. The main limitation was the absence of a reference standard for comparing the results with CTA or DSA. However, this study focused on screening of RAS by coronal or axial image acquisition. Second, we have performed with preset value of TI = 1500 ms in all 128 subjects without considering the inflow effect in aging population. We expect that elderly has a tendency of having a slower blood flow of aorta to renal arteries. Third, the coronal slice direction is anterior–posterior of the body to observe aorta, renal arteries, and iliac bifurcation, whereas the axial scan requires more slice coverage in the *Z*-direction to observe aorta, renal arteries, and iliac bifurcation. Therefore, having the same resolution as the coronal scan obviously costs a lot longer scan time in the axial scan. Lastly, image registration was not performed during image processing.

In conclusion, this study showed that coronal image acquisition provides superior contrast between the renal arteries and the background and allows more persistent visualization of renal artery branches than axial acquisition in non-contrast-enhanced MRA using flow-in bSSFP with Time-SLIP. Therefore, the first-line screening of renal non-contrast-enhanced MRA can be performed with coronal acquisition, which permits a wider view of the abdominal aorta and renal arteries, and facilitates an understanding of the overall relationship of the surrounding arteries, such as the hepatic artery.
